# CD4^+^CD25^hi^CD127^low^ Regulatory T Cells Are Increased in Oral Squamous Cell Carcinoma Patients

**DOI:** 10.1371/journal.pone.0103975

**Published:** 2014-08-25

**Authors:** Kue Peng Lim, Nicole Ai Leng Chun, Siti Mazlipah Ismail, Mannil Thomas Abraham, Mohd Nury Yusoff, Rosnah Binti Zain, Wei Cheong Ngeow, Sathibalan Ponniah, Sok Ching Cheong

**Affiliations:** 1 Oral Cancer Research Team, Cancer Research Initiatives Foundation (CARIF), 2^nd^ Floor Outpatient Centre, Subang Jaya, Selangor, Malaysia; 2 Oral Cancer Research and Coordinating Centre, Faculty of Dentistry, University of Malaya, Kuala Lumpur, Malaysia; 3 Department of Oro-Maxillofacial Surgery and Medical Sciences, Faculty of Dentistry, University of Malaya, Kuala Lumpur, Malaysia; 4 Department of Oral & Maxillofacial Surgery, Tengku Ampuan Rahimah Hospital, Klang, Malaysia; 5 Cancer Vaccine Development Program, Uniformed Services University of the Health Sciences, Bethesda, Maryland, United States of America; Pavillon Kirmisson, France

## Abstract

Regulatory T cells (Tregs), a subset of CD4^+^ T cells plays a pivotal role in regulating the immune system. An increase in Treg numbers enables cancer progression by dampening the immune system and allowing tumor cells to evade immune detection and destruction. An increase in Treg numbers and expression of inhibitory cytokines including TGF-β and IL-10 are mechanisms by which Tregs exert their immune suppressive function. However, the presence of Tregs and inhibitory cytokines in oral cancer patients is still unclear. In this study, the presence of circulating Tregs in 39 oral cancer patients and 24 healthy donors was examined by studying the presence of the CD4^+^CD25^hi^CD127^low^ cell population in their peripheral blood mononuclear cells using flow cytometry. Serum levels of TGF-β and IL-10 were measured by ELISA. T cell subsets of OSCC patients were found to differ significantly from healthy donors where a decrease in CD8^+^ cytotoxic T cells and an increase in Tregs (CD4^+^CD25^hi^CD127^low^) were observed. Further, the ratio of CD8^+^ T cells/Tregs was also decreased in patients compared to healthy donors. The presence of Tregs was accompanied by a decrease in IL-10 but not TGF-β secretion in OSCC patients when compared to donors; in addition, the analysis also revealed that an increased presence of Tregs was accompanied by better patient survival. Amongst OSCC patients, smokers had significantly higher levels of TGF-β. It is apparent that the immune system is compromised in OSCC patients and the characterization of the Treg subpopulation could form a basis for improving our understanding of the perturbations in the immune system that occur during OSCC tumorigenesis.

## Introduction

The inability of the immune system to eradicate established tumors is a well-recognized hallmark of cancer [1], [2]. Tumors can employ numerous mechanisms to suppress host immunity and in particular, many studies have focused on the role of regulatory T cells (Tregs) in helping tumors evade immune surveillance [3]–[6]. Whilst Tregs are important regulators of immune-mediated inflammation, they have been demonstrated to be equally critical as mediators of active immune evasion; the depletion of these cells can improve endogenous anti-tumor immunity and efficacy of immunotherapy [7], [8]. This realization has resulted in the development of strategies to target the immunosuppressive arm of the immune system [9], [10].

Treg-mediated suppression can occur through several mechanisms, either by cell-cell contact or by secretion of cytokines (TGF-β and IL-10) [11]. The involvement of Tregs in tumor progression in patients with lung, head and neck, prostate, and breast cancers has been demonstrated, where there are increased levels of these cells in the peripheral blood [12]–[15]. Further, levels of IL-10 which mediates immune suppression by down-regulating MHC class I expression or by inhibiting T cell activation were shown to be increased in many types of cancers including melanoma, head and neck, pancreatic, gastric, lung, and breast [16]–[23]. TGF-β is a potent suppressor of the immune system and it inhibits the secretion of immunoglobulin (Ig) M, IgG1, IgG2a and IgG3 [24]. Neutralizing antibodies to TGF-β have been shown to reverse Treg-mediated suppression in inflammatory bowel disease (IBD) in mice and thyroiditis in rats [25], [26]. In the main, the presence of Tregs has been associated with poor disease outcome, however, contradictory reports exists [12], [13], [15], [27]–[34]. The discrepancies happen in part because of the multiple markers that are used to identify the Treg population. Markers delineating these cells include CD25, cytotoxic T lymphocyte-associated antigen 4 (CTLA-4), glucocorticoid-induced tumor necrosis factor receptor family-related gene (GITR), lymphocyte activation gene-3 (LAG-3), CD127 and forkhead / winged-helix transcription factor box P3 (FOXP3) [26], [35]–[39]. As many of these markers are also markers of T cell activation [40], CD4^+^CD25^+^FOXP3^+^ and CD4^+^CD25^+^CD127^low^ remain the most well accepted markers for the identification of Tregs, as cells with these phenotypes are immunosuppressive [41]–[43].

Head and neck cancers pose a significant global burden [44], [45] and oral cancer, a subset of head and neck cancers occurs in about 400,000 individuals world-wide, and contribute to more than 222,000 deaths annually [44], [46]. Moreover 50% of oral cancer patients suffer from disease recurrence or secondary tumors [47]–[49]. As we begin to identify key genetic drivers through the compilation of gene expression, chromosomal copy number and sequencing data for many types of solid tumors [50], [51], the development of novel treatments (especially immunotherapy) to target some of the mutations arising from these genetic events is currently possible [52]. However, the presence of immune suppression in patients could still hinder the success of these novel treatment modalities. In this study, the immunological status of oral cancer patients was characterized by looking at their lymphocyte subsets, and the levels of IL-10 and TGF-β with the hypothesis that OSCC patients would have a compromised immune system with an increased presence of Tregs and levels of IL-10 and TGF-β. A significant increase in CD4^+^CD25^hi^CD127^low^ sub-population of Tregs in OSCC patients and a novel subpopulation of CD4^+^CD25^hi^ that completely lacked of CD127 expression was found in OSCC patients. Further, the presence of regulatory T cells was accompanied by a decrease in IL-10 but not TGF-β secretion. Our results show that the level of Tregs differs between OSCC patients and normal individuals suggesting that these cells may play a major role in oral carcinogenesis.

## Materials and Methods

### Peripheral Blood Mononuclear Cell (PBMC) isolation

Thirty-nine OSCC patients (no prior treatment) and 24 healthy donors were included in this study. Healthy donors were recruited from relatives or friends who accompanied the patient and at blood donation drives. All individuals were briefed regarding the project prior to obtaining written consent; in the event of an individual who was unable to provide written consent due to the inability to write, written consent was either obtained in the form of a thumb print or from the next of kin. All consent documents were filed and kept for future reference in the Oral Cancer Research & Coordinating Centre (OCRCC), Faculty of Dentistry, University of Malaya. All relevant documents and procedures were approved by the institutional review board of the Faculty of Dentistry Medical Ethics Committee, University Malaya (Medical ethics number: DF OS0910/0049(L). Eight to thirty milliliters of peripheral blood was collected from each of these individuals in BD Vacutainer CPT tubes (Becton Dickinson, NJ, USA). Peripheral blood mononuclear cells (PBMC) were isolated from the peripheral blood, washed in Hanks balanced salt solution (HBSS; Gibco, Life Technologies, California, USA) and re-suspended in complete culture medium [Roswell Park Memorial Institute Medium (RPMI; Gibco, Life Technologies, California, USA) supplemented with 5% heat-inactivated human AB serum (Gemini Bio-Product, California, USA) and 1× penicillin/streptomycin (Gibco, Life Technologies, California, USA)]. PBMC (1×10^6^)were used for the assays as described below.

### Identifying circulating T cell subsets

**Figure 1:**
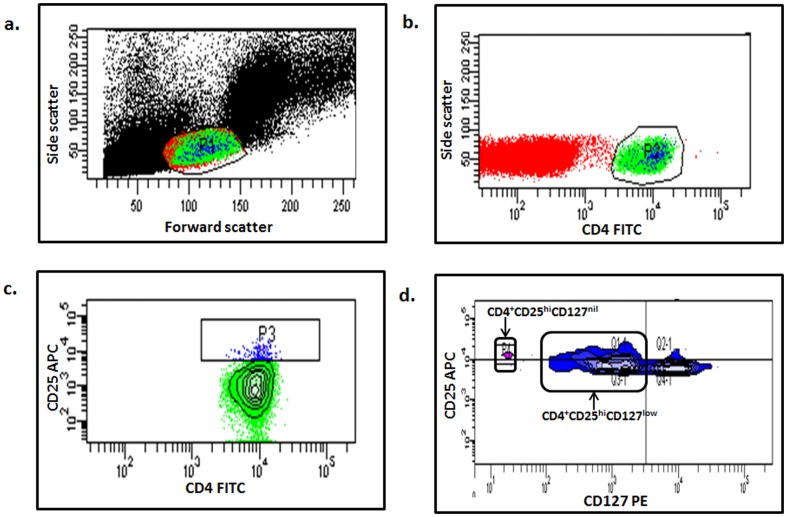
Gating used for the identification of circulating T cell subsets in human. Multiparameter flow cytometry analyses of CD4^+^CD25^hi^, CD4^+^CD25^hi^CD127^low^ and CD4^+^CD25^hi^CD127^nil^ regulatory T cells subsets. Gates were set on (a) T lymphocytes (based on forward and side scatter), (b) CD4^+^ T cells, (c) CD4^+^CD25^hi^ and (d) CD4^+^CD25^hi^CD127^low^ and CD4^+^CD25^hi^CD127^nil^.

The presence of Tregs was determined in 35 OSCC patients and 14 healthy donors by measuring the following markers: CD4 (BD Biosciences-Pharmingen, California, USA), CD25 (e-Bioscience, CA, USA) and CD127 (BD Biosciences-Pharmingen, California, USA). Briefly, 5×10^5^ fresh PBMCs were incubated with specific antibodies conjugated with fluorochrome (0.42 µg/ml of CD25-APC, 0.33 µg/ml of CD4-FITC and 0.33 µg/ml of CD127-PE) for 30 minutes at 4°C. The cells were then washed using phosphate buffered saline (PBS), resuspended in Pharmingen Stain Buffer (PSB) and analyzed using a flow cytometer (FACSCalibur, BD Biosciences, California, USA). The presence of Tregs was then analyzed using FACSDiva analysis software (BD Biosciences, California, USA). Briefly, 50,000–100,000 cells were collected and lymphocyte population was gated based on cell size (Figure 1a) and the presence of helper T cells (CD4^+^), CD4^+^CD25^hi^ and CD4^+^CD25^hi^CD127^low^ cells were further determined from the CD4^+^ cell population (Figure 1b, c, d). In addition, the presence of CD4^+^CD25^hi^CD127^nil^ cells were also identified (Figure 1d). Similarly, CD4^+^ and CD8^+^ T cell subsets were detected with TCR-APC (Miltenyi Biotec, Surrey, UK), CD4-FITC (BD Biosciences-Pharmingen, California, USA) and CD8-PE (Invitrogen, NY, USA) antibodies and stained as described above in 35 OSCC patients, 9 healthy donors and 27 OSCC patients and 9 healthy donors respectively.

### Measurement of IL-10 and TGF-β by Enzyme-linked Immunosorbent Assay (ELISA)

The levels of two cytokines (IL-10 and TGF-β) which are known to be involved in the immunosuppressive function of Tregs were analyzed in 30 OSCC patients and 16 healthy donors using ELISA, according to the manufacturer's protocol (Invitrogen, New York, USA). Prior to analyzing TGF-β levels, 100 µl of serum was activated by adding 4 µl of 1N HCl and incubated at room temperature for 15 minutes and neutralized using 3 µl 1N NaOH. The activated serum was then diluted 4× with assay buffer and dispensed into 96 well plates which were pre-coated with TGF-β antibody, this was followed by the addition of the detection antibody and streptavidin-HRP conjugate. The specific binding was visualized after adding the substrate solution. A standard curve (log-log curve fit) was generated using respective recombinant human cytokine and the absolute concentration of cytokine in the serum was determined from this curve. Detection of IL-10 levels was performed as described above without the serum activation procedure.

### Statistical analysis

All statistical analyses were performed using the statistical software package SPSS 16 (SPSS Inc., Chicago, IL, USA) and GraphPad Prism software (GraphPad Inc., CA, USA). Independent t-test was used to compare the differences of immune cells/cytokines levels in patients and healthy donors. The correlation between immune cells subsets and cytokines levels with clinico-pathological factors was estimated by Fisher's exact test. Kaplan-Meier survival analysis was used to correlate survival rates with the level of CD4^+^CD25^hi^CD127^low^ regulatory T cells, the survival probability differences were compared by log-rank test. A *p-*value of <0.05 was considered to be statistically significant.

## Results

### Demographic and clinical characteristics of OSCC patients

The demographic and clinical characteristics of the patients included in this study are tabulated in Table 1. The mean age of the OSCC patients was 58±13 years (range: 26–76 years). The ethnic distribution of the patients was representative of the OSCC patient population in Malaysia where the majority of the patients were of Indian ethnicity. More than 50% of the patients were diagnosed with late stage disease (stage III & IV; Table 1). The mean age of healthy donors was 32±8 years (range 24–49 years), but other demographic information on these individuals were not available. Survival information was available for 32 of 39 patients, in which the length of follow up ranged from 2 to 85 months until May of 2013, with a median survival of 24 months.

**Table:** **Table 1.** Patients' demographic data.

	Variables		OSCC	
Age (year)		Mean ± SD: 58±13	n	%
Gender		Male	8	21
		Female	31	79
Ethnic		Malay	3	8
		Chinese	4	10
		Indian	31	79
		Others*	1	3
Habits		Ever Smoker	6	15
		Ever Drinker	5	13
		Ever Chewer	23	59
		No habit	10	26
Stage		Early (stage I & II)	13	33
		Late (stage III & IV)	23	59
		Data not available	3	8
Disease sites		Cheek	16	41
		Tongue	10	26
		Gingiva	5	13
		Others**	7	18
		Data not available	1	2

*Others: Indigenous population.

** Others: Lip, palate, floor of mouth.

### Comparison of circulating T cell subsets in OSCC patients and healthy donors

**Figure 2:**
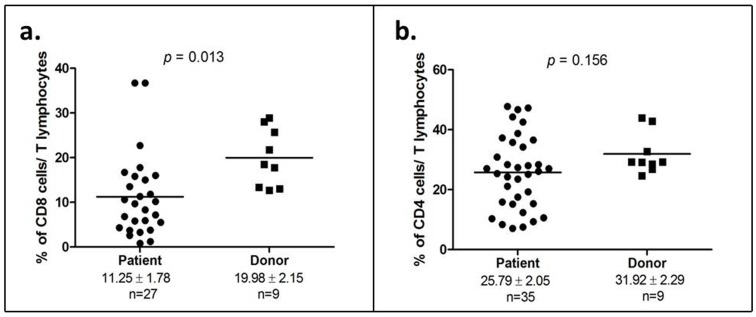
Reduction of cytotoxic T cells (CD8^+^) is a characteristic of OSCC patients. (a) Percentage of cytotoxic T lymphocytes (CD8^+^) in both OSCC patients and healthy donors; (b) Percentage of helper T cells (CD4^+^) in both OSCC patients and healthy donors. Each symbol represents an individual and the narrow bar represents the mean percentage of the specific cell population.

T lymphocyte subpopulations in OSCC patients and healthy donors were analyzed using flow cytometry. No difference was found in the average percentage of T lymphocytes between OSCC patients and healthy donors (data not shown). However, the analysis of sub-populations of T lymphocytes demonstrated a significant reduction in the cytotoxic T cells population (CD8^+^) in OSCC patients compared to healthy donors (11.25±1.78 vs 19.98±2.15; *p* = 0.013; Figure 2a). Further, a decrease in helper T cells (CD4^+^) count was also seen in OSCC patients compared to healthy donors but this difference was not statistically significant (25.79±2.05 vs 31.92±2.29; *p* = 0.156; Figure 2b).

**Figure 3:**
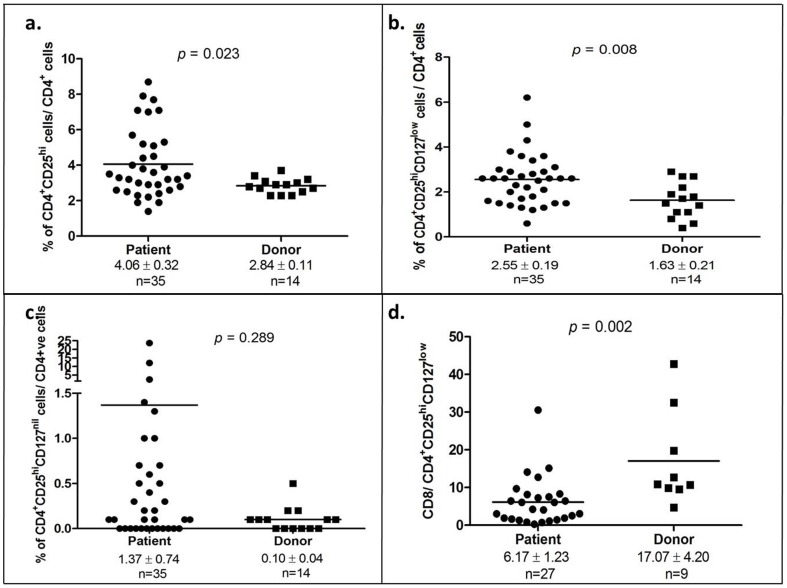
CD4^+^CD25^hi^CD127^low^ regulatory T cells were up-regulated in OSCC patients. (a) Percentage of CD4^+^CD25^hi^ T cells amongst CD4^+^T cells in OSCC patients and healthy donors; (b) Percentage of CD4^+^CD25^hi^CD127^low^ T cells amongst CD4^+^ T cells in OSCC patients and healthy donors; (c) Percentage of CD4^+^CD25^hi^ CD127^nil^ T cells amongst CD4^+^ T cells in OSCC patients and healthy donors; (d) Ratio of CD8 T cells/CD4^+^CD25^hi^CD127^low^ T cells in OSCC patients and healthy donors; Each symbol represents an individual person and the narrow bar represents the mean percentage of the specific cell population.

The percentage of CD4^+^CD25^hi^ was significantly elevated in OSCC patients compared to healthy donors (4.06±0.32 vs 2.84±0.11; *p* = 0.023; Figure 3a). Notably, the expanded subset of the CD4^+^CD25^hi^ cells, the CD4^+^CD25^hi^ CD127^low^ T cells (which are indicative of defined regulatory T cells) was prominently higher in OSCC patients compared to healthy donors (2.55±0.19 vs 1.63±0.21; *p* = 0.008; Figure 3b). Of note, another population of cells that totally lacked the expression of CD127 was detected and designated here as the CD4^+^CD25^hi^CD127^nil^ population; this subpopulation of CD4^+^CD25^hi^ cells demonstrated a diverse presence in patients ranging from 0%–23.7% of the CD4^+^ T cell population, whilst this diversity was not observed in healthy donors (0%–0.4% of CD4^+^ T cells). However, when comparing the average levels of CD4^+^CD25^hi^CD127^nil^ levels, no significant difference was observed between OSCC patients and healthy donors (1.37±0.74 vs 0.10±0.04; *p* = 0.289; Figure 3c). Given that a significant reduction in CD8^+^ T cell population and a prominent increment of the of CD4^+^CD25^hi^ CD127^low^ regulatory T cells were observed, the ratio of CD8^+^ T cell to CD4^+^CD25^hi^ CD127^low^ regulatory T cells was further determined; OSCC patients demonstrated a 3-fold decrease in the ratio as compared to healthy donors (6.17±1.23 vs 17.07±4.20; *p* = 0.002).

### The presence of TGF-β and IL-10 in the serum of OSCC patients and healthy donors

**Figure 4:**
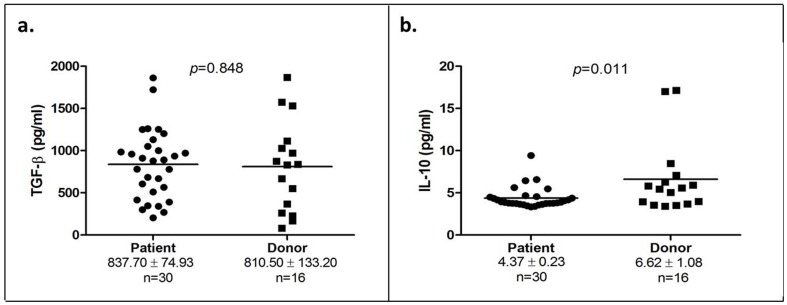
TGF-β and IL-10 levels in OSCC patients and healthy donors. (a) TGF-β concentration of 30 OSCC patients and 16 healthy donors; (b) IL-10 concentration of 30 OSCC patients and 16 healthy donors. Each symbol represents an individual person and the narrow bar represents the mean percentage of the specific cell population.

We did not find any difference in the level of TGF-β in OSCC patients compared to healthy donors (837.704±74.93 pg/ml vs 810.50±133.20 pg/ml; *p* = 0.848; Figure 4a). However, our results exhibited a significant decrease in the IL-10 level in OSCC patients as compared to healthy individuals (4.37±0.23 pg/ml vs 6.62±1.08 pg/ml samples; *p = *0.011 Figure 4b).

### Correlation of circulating T cell subsets and cytokines with patient clinico-pathological factors

We analyzed if there were any correlation between of the percentage of Tregs (CD4^+^CD25^hi^CD127^low^) and the levels of TGF-β/IL-10 with patient's clinico-pathological parameters. Our data showed that OSCC patients who smoke have significantly higher TGF-β levels compared to non-smokers (*p* = 0.001; Table 2A) but no correlation between IL-10 levels with patients' clinico-pathological parameters was observed (Table 2B). Furthermore, patients who were at early stages of oral cancer had higher levels CD4^+^CD25^hi^CD127^low^ regulatory T cells (*p* = 0.027, Table 3A) but this correlation was not seen for the CD8^+^ T cells/Tregs ratio (Table 3B). In addition, no association between the levels of Tregs, IL-10 and TGF-β was found (data not shown).

**Table:** **Table 2.** The correlation between TGF-β (A) and IL-10 (B) levels with patient clinico-pathological factors.

A			
Factor	N	TGF-β	*p* value
		Mean ± SD	
**Staging**	29		
Early	10	882.50±562.78	
Late	19	822.93±331.82	0.721
**Ever chewer**	30		
Yes	18	818.88±391.54	
No	12	865.87±453.55	0.765
**Ever smoker**	30		
Yes	5	1339.58±296.05	
No	25	737.30±354.90	0.001
**Ever drinker**	30		
Yes	4	955.53±385.28	
No	26	819.55±418.35	0.547
**Mean Age**	30		
≥58	22	795.83±402.34	
<58	8	953.76±437.67	0.363

B			
Factor	N	IL-10	*p* value
		Mean ± SD	
**Staging**	29		
Early	10	4.17±0.62	
Late	19	4.50±1.52	0.515
**Ever chewer**	30		
Yes	18	4.47±1.51	
No	12	4.23±0.79	0.626
**Ever smoker**	30		
Yes	5	4.48±1.21	
No	25	4.35±1.29	0.833
**Ever drinker**	30		
Yes	4	4.08±0.34	
No	26	4.41±1.35	0.630
**Mean Age**	30		
≥58	22	4.21±0.88	
<58	8	4.81±1.98	0.255

**Table:** **Table 3.** The correlation between specific T cell populations and patient clinico-pathological factors.

A			
Factor	N	CD4+CD25hiCD127low	*p* value
		Mean ± SD	
**Staging**	33		
Early	12	3.08±1.41	
Late	21	2.01±0.80	0.027
**Ever chewer**	35		
Yes	19	2.37±1.16	
No	16	2.78±1.10	0.299
**Ever smoker**	35		
Yes	6	2.73±1.04	
No	29	2.52±1.17	0.678
**Ever drinker**	35		
Yes	4	2.45±0.97	
No	31	2.57±1.17	0.849
**Mean Age**	35		
≥58	22	2.53±1.26	
<58	13	2.59±0.93	0.882

B			
Factor	N	CD8+ T cells/regulatory T cells	*p* value
		Mean ± SD	
**Staging**	27		
Early	8	4.42±2.89	
Late	19	6.90±7.36	0.369
**Ever chewer**	27		
Yes	16	6.73±4.52	
No	11	5.35±8.66	0.592
**Ever smoker**	27		
Yes	6	7.42±11.42	
No	21	5.81±4.50	0.748
**Ever drinker**	27		
Yes	4	4.06±1.71	
No	23	6.53±6.87	0.489
**Mean Age**	27		
≥58	19	6.75±7.19	
<58	8	4.77±4.07	0.473

### High levels of CD4^+^CD25^hi^CD127^low^ Tregs is associated with better survival

**Figure 5:**
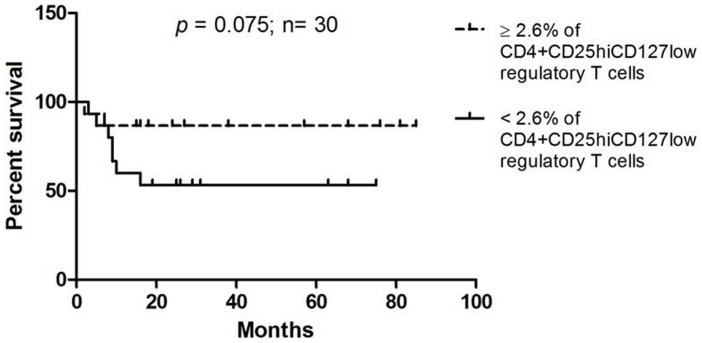
Disease-specific Kaplan-Meier survival estimates based on the levels of CD4^+^CD25^hi^CD127^low^ regulatory T cells.

To evaluate the correlation of the presence of CD4^+^CD25^hi^CD127^low^ regulatory T cells to patient survival, we used the mean percentage of CD4^+^CD25^hi^CD127^low^ regulatory T cells (2.6%) of all patients as cut off point and divided patients into those who have equal or more than 2.6% of CD4^+^CD25^hi^CD127^low^ regulatory T cells and those who have less than 2.6% of CD4^+^CD25^hi^CD127^low^ regulatory T cells. Using Kaplan-Meier analysis, patients who had higher levels of CD4^+^CD25^hi^CD127^low^ regulatory T cells were found to have better survival probability compared to patients with lower CD4^+^CD25^hi^CD127^low^ regulatory T cells, however this did not reach statistical significance (*p* = 0.075; Figure 5).

## Discussion

The homeostasis between immune activation and immune suppression is important in preventing tumorigenesis. The immune system functions either to eliminate cancer cells or to keep cancer cells in check by maintaining an equilibrium; if there is a malfunction in the immune system, immune escape may happen which then allows cancer cells to grow into clinically apparent tumors [53]. The mechanisms by which tumor escapes immune-surveillance include deregulation of the function of Tregs and/or immune-regulatory cytokines.

In our study, the percentage of T lymphocytes in both the OSCC patients and healthy donors were not significantly different. Previous reports have demonstrated a reduction of T lymphocytes in cancer patients, the main difference between our study and these may lie in the fact that in our study, treatment naïve patients were recruited whereas most other studies included post-treated cancer patients who had undergone chemo/radiotherapy which could result in lymphodepletion [54], [55]. Looking at the subsets of T cells, a reduction in the CD8^+^ T cell population was observed, which suggests a compromised immune system, as CD8^+^ T cells are involved in adaptive immunity that is responsible for eliminating tumor cells when the cancer develops [56]. This data is consistent with a previous report where a reduction in the CD8^+^ T cell population was also observed in head and neck cancer patients [57].

Tregs have been reported to be increased in the peripheral blood of various cancers [7], [13], [58]–[60]. While the levels of circulating Tregs have initially been reported to be increased in head and neck cancers [60], [61], emerging data showed no differences in the Treg numbers between head and neck cancer patients and healthy controls [43], [62]. These discrepancies are likely due to the differences in patient cohorts where a variety of anatomical sub-sites were analyzed, and the utilization of different markers in defining the Treg cell populations. While we observed a significant increase in the Treg cell populations in OSCC patients compared to healthy donors, Drennan et al. found no difference despite using identical markers in identifying Tregs (CD4^+^CD25^high^CD127^low/−^) [43]. The possible reason for this discrepancy is that the current study analyzed OSCC patients while Drennan and colleagues conducted their study purely on patients with laryngeal and oropharyngeal cancers. Differences in OSCC and oropharyngeal are well established, and the most apparent is the involvement of HPV where up to 60% of oropharyngeal cancer have been reported to be HPV positive [63]. By contrast, the prevalence of HPV infection in OSCC is low [64] and emerging data from our laboratory indicate that the prevalence of HPV infection in Malaysia OSCC patients is in the range of 1.5% (TG Kallarakkal et al; unpublished data), suggesting that this could be one of the reasons for the discrepancy that is observed. In our study, high levels of CD4^+^CD25^hi^CD127^low^ cells were found to be significantly increased in early stage OSCC patients compared to late stage patients and further found to trend with better patient survival. This seemingly contradictory observation of a better prognosis associated with higher levels of Tregs could be explained by their role in reducing tumor-specific immune-mediated inflammation which has been shown to drive the progression of tumors in certain cases [65]–[67]. This finding is in line with other studies demonstrating that the presence of Tregs correlated with better loco-regional control in head and neck cancer and the presence of Treg is an indicator of better overall survival in Hodgkin's lymphoma [12], [68]. Indeed, adoptive transfer of Tregs in mice has been shown to suppress colitis-associated colon cancer and intestinal adenomas [69], [70] and this could likely be due to the ability of Tregs in reducing the effects of chronic inflammation by down-regulating the expression of cyclooxygenase-2 and pro-inflammatory cytokines including TNF-α and IFN-γ. This effect on other solid tumors remains to be tested. Further, the CD8^+^ T cell/Treg ratio has been shown to be significantly associated with patient survival [5], [71], although a meaningful reduction of this ratio in OSCC patients was observed, correlation with patient survival was not significant. In addition a subpopulation where CD127 is not expressed (CD4^+^CD25^hi^CD127^nil^) was detected, this population has not yet been reported and the significance and function if any, of this cell population remains unclear.

In addition to the up-regulation of Tregs, the secretion of immune regulatory cytokines particularly TGF-β and IL-10 is also known to be a mechanism of immune suppression [72], [73]. The increase expression of IL-10 in head and neck squamous cell carcinoma patients remains controversial. Several studies have suggested that patients with advanced head and neck cancer have elevated serum levels of IL-10 and this finding is associated with poor prognosis [74], [75]. However, others failed to detect a differential expression of IL-10 in these patients [76], [77]. The main reason for these could be the inherent differences in tumours from different sites of the head and neck where patients with oral cavity cancers are more likely to have undetectable levels of IL-10 compared to other sub-sites [74]. In the present study, the IL-10 levels were decreased or undetectable in OSCC patients and were not correlated with any patient's demographic information. This is consistent with the findings of Green et al. where IL-10 levels were not associated with any clinicopathological parameters nor with overall patient survival [78]. As for the serum levels of TGF-β, both patients and healthy donors were found to have similar levels of this cytokine. However, further analysis showed that smokers had significantly higher levels of TGF-β compared with non-smokers who incidentally were mainly betel quid chewers. TGF-β is known to have complex and contradictory growth effects on cancer cells [79] but the relationship between smoking and increase in TGF-β levels is still not well understood and warrants further investigation. Perhaps the lack of TGF-β and IL-10 is not too surprising as others have demonstrated that in head and neck cancers, Tregs that primarily secrete IL-10 and TGF-β are those within the population of tumor infiltrating lymphocytes and are not those in circulation [80].

In summary, the current findings are consistent with other studies, OSCC patients have a compromised immune status where the CD8^+^ T cell levels are reduced and the Treg population is elevated. Within the OSCC patient cohort however, higher levels of Treg were associated with better survival. A new group of T cells that is CD4^+^CD25^hi^CD127^nil^ have also been demonstrated which has not been reported previously. The presence of this population of cells particularly within the OSCC patients warrants further investigation.
